# Description of three new species of Apatelodidae from the southern neotropical region (Lepidoptera, Bombycoidea)

**DOI:** 10.3897/zookeys.788.25323

**Published:** 2018-10-08

**Authors:** Daniel Herbin, Hernán Mario Beccacece

**Affiliations:** 1 7; 2 , Le Clos de Lutché, F-31380 Garidech, France; 3 Universidad Nacional de Córdoba, Facultad de Ciencias Exactas, Físicas y Naturales, Centro de Investigaciones Entomológicas de Córdoba; Av. Vélez Sársfield 1611, Córdoba, Argentina

**Keywords:** *
Apatelodes
*, Neotropical fauna, *
Quentalia
*, taxonomy

## Abstract

Three new species of Apatelodidae are described from Argentina, Bolivia, Brazil, and Paraguay: *Apatelodesnavarroi***sp. n.**, *Apatelodeschalupae***sp. n.**, and *Apatelodesulfi***sp. n.**, and are figured with their genitalia. Detailed examination of primary types leads to the establishment of a new synonymy: *A.florisa* Schaus, 1929 = *A.schreiteri* Schaus, 1924, **syn. n.**, and a revised status for another Apatelodidae species previously misplaced in the Bombycidae: *Apatelodesbrunnea* (Dognin, 1916), **comb. n.**

## Introduction

The adults of the American silkworm moths (Lepidoptera, Bombycoidea, Apatelodidae) are small to medium size with earthen tones. The larvae are exposed feeders on trees and shrubs and several species are polyphagous (Gillott 2005). This family is mainly Neotropical with a few representatives in North America (Kitching et al. 2018).

An extensive revision of Apatelodidae was published by Draudt (1929). More recently, a checklist was issued by Becker in Heppner (1996), and a Bombycoidea checklist by Kitching et al. (2018). The status of Apatelodidae as a family belonging to the Bombycoidea has been confirmed by morphological and molecular studies (Lemaire and Minet 1998, Zwick et al. 2011).

Recent taxonomic studies of Apatelodidae suggested that this family contains more species than previously thought: Beutelspacher Baigts (1984), Wagner and Knudson (2014), Herbin (2015, 2017), Herbin and Monzón Sierra (2015), Herbin and Mielke (2018), and ongoing taxonomic and phylogenetic work will certainly reveal many more.

Within the Apatelodidae, the most diverse genus is *Apatelodes* Packard, 1864, which contains 115 species of the total of 214 species in the family, as per the most recent report by Kitching et al. (2018). The caterpillars of *Apatelodes* are generally hairy, with long hairs, and pupate under ground. A large variety of food plants seem to be used by species in the genus, as found by extensive rearing conducted in Costa Rica, Guanacaste, and reported on a dedicated Internet site by Janzen and Hallwachs (2009). Hosts of *Apatelodes* include plants from the families: Malvaceae, Chrysobalanaceae, Myrtaceae, Fabaceae, Salicaceae, Meliaceae, Annonaceae, Asteraceae, Piperaceae, Amaranthaceae, Convolvulaceae, Verbenaceae, and Bignoniaceae. An *Apatelodes* species from Colombia has recently been reared in the laboratory in Europe, and accepted a *Salix* Linneaus sp. (Salicaceae) as a replacement food plant (Herbin unpublished), and some other *Apatelodes* species are reported as pests of banana trees (*Musa* Linneaus, Musaceae) in Venezuela (Dominguez et al. 2002).

In this study, three new species of Apatelodidae from Argentina and neighboring countries (Brazil, Bolivia and Paraguay) are described in *Apatelodes* based on their habitus, genitalia, and DNA barcoding.

## Materials and methods

Materials and methods are as per Herbin (2017) and Herbin and Mielke (2018).

Figures were manipulated with Adobe Photoshop CS4. Green labels in figures relate to a voucher number in CDH (see below for collection abbreviations). White labels with the format “BC-Her####” relate to barcode reference numbers from specimens in CDH. All other labels shown belong to the holotype.

All species treated here were subjected to DNA analysis using the DNA barcode region of the mitochondrial COI gene in BOLD (Barcode of Life Data System: http://www.boldsystems.org, see also Ratnasingham and Hebert (2007)). Sequences were aligned using the tools provided in BOLD (BOLD Aligner: Amino Acid Based HMM), exported in fasta format, and imported in MEGA6 (Tamura et al. 2013). The evolutionary history of the taxa was inferred using the Neighbor-Joining method (Saitou and Nei 1987). The phenograms are drawn to scale, with branch lengths (next to the branches) in the same units as the evolutionary distances used to infer the Neighbor-Joining tree. The evolutionary distances were computed using the Kimura 2-distances (Kimura 1980) and measure the number of base substitutions per site. All codon positions were included and all positions containing gaps and missing data were excluded.

Description of colors in the descriptions refers to the RAL color standard, see https://www.ral-farben.de/en/home/.

### Abbreviations

**CDH** Collection Daniel Herbin, Garidech, France

**IFML** Instituto Fundación Miguel Lillo, Tucumán, Argentina

**MFN** Museum für Naturkunde, Leibniz-Institut für Evolutions- und Biodiversitätsforschung, Berlin, Germany

**MHNG** Museum d’Histoire naturelle de Genève, Genève, Suisse

**MNHN** Muséum national d’Histoire naturelle, Paris, France

**NHMUK** Natural History Museum, London, U.K.

**USNM** National Museum of Natural History [formerly United States National Museum], Washington, D.C., USA

## Taxonomy

### Apatelodes navarroi sp. n.

Taxon classificationAnimaliaLepidopteraBombycoidea

http://zoobank.org/360E2A19-1396-438A-BB6D-BA93C28BCD89

Figs 1 , 2 , 3 , 4

#### Types.

**Figure 1.**
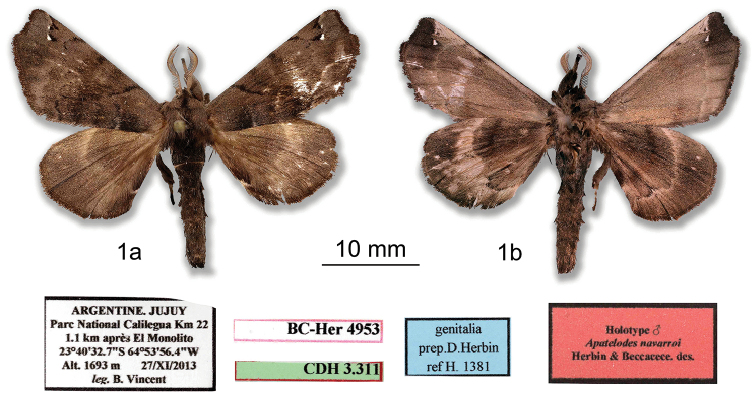
*Apatelodesnavarroi* sp. n. male holotype: **a** Dorsal view **b** Ventral view.

**Figure 2.**
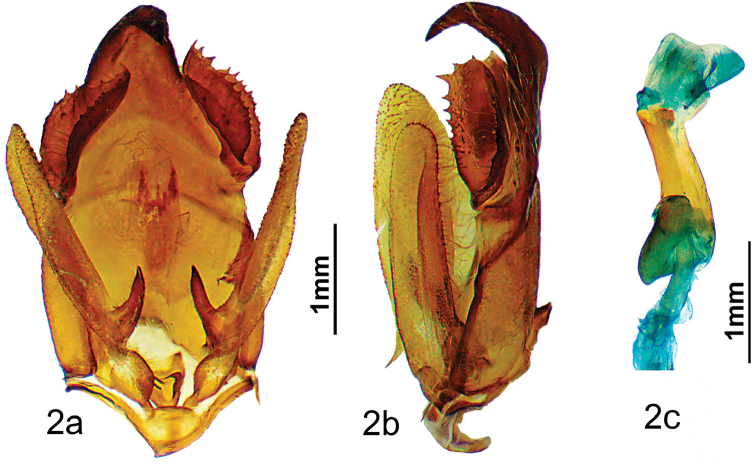
*Apatelodesnavarroi* sp. n. male holotype genitalia: **a** Ventral view **b** Lateral view **c** Phallus lateral view.

***Holotype male.* ARGENTINA: Jujuy**: Parc National Calilegua Km 22, 1.1 km après El Monolito, 23°40'32.7"S; 64°53'56.4"W, Alt. 1693m, 27/XI/2013, *leg.* B. Vincent / genitalia prep. D. Herbin ref. H1381 / HOLOTYPE ♂ *Apatelodesnavarroi* Herbin & Beccacece. des. / CDH 3.311 / BC-Her4953. (Figs 1, 2). *In*MNHN.

**Figure 3.**
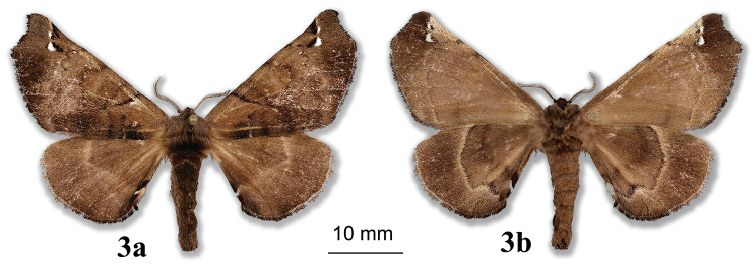
*Apatelodesnavarroi* sp. n. female paratype: **a** Dorsal view **b** Ventral view.

***Paratypes.*** 1 female. **ARGENTINA: Jujuy**: Parc National Calilegua Km 21, El Monolito, 23°40'56.1"S; 64°54'06"W, Alt. 1723m, 01/XII/2013, *leg.* B. Vincent / genitalia prep. D. Herbin ref. H1386 / CDH 3.322. (Fig. 3). *In*CDH.

#### Etymology.

*Apatelodesnavarroi* sp. n. is named after the late Dr. Fernando Navarro (IFML), who directed the expedition that enabled the discovery of this new species and the following one.

#### Diagnosis.

*Apatelodesnavarroi* sp. n. belongs to a small group of Apatelodidae showing very developed socii. This group includes *A.hierax* Dognin, 1924, *A.schreiteri* Schaus, 1924, *A.florisa* Schaus, 1939, *A.zikani* Draudt, 1929, and *A.combi* Herbin, 2015. *Apatelodesnavarroi* sp. n. is similar to *A.florisa* and *A.schreiteri*, both described from Argentina, Tucumán (see taxonomical notes hereafter). *Apatelodesnavarroi* sp. n. is easily distinguished by its very dark brown color, its greater size, the elongated shape of the forewings, and the slightly concave termen of the forewing below apex. The male genitalia exhibit large socii with well-developed teeth.

#### Description.

***Male.****Antennae*: antennae bipectinate to the tip. Scape, pedicel, and antennomeres beige, rami brown. *Head*: brown, labial palpi thick, brown, slightly ascending and reaching beyond the front. Eyes naked, dark brown. *Thorax*: coloration terra brown (RAL8028) with a median longitudinal black line. *Legs*: coloration as for thorax, with tibia appearing very thick as covered by long light brown hair like scales. Femora densely pilose beige with dark brown scales; *Abdomen*: sepia brown (RAL8014). *Forewing dorsum*: Forewing length: 17 mm, wingspan: 34 mm. Triangular, apex acute, outer margin slightly concave below apex. Coloration terra brown (RAL8028), antemedial line black, a basal rectangular black mark in antemedial area, postmedial line wavy, black. Two small hyaline spots near apex and costa: one tiny spot near costa, bordered proximally with small black triangular mark, second spot posterior to first slightly larger, triangular, and bordered proximally with black scales. Between costa and first tiny hyaline spot, a black comma marking present. *Forewing ventrum*: Similar to dorsum, but lighter in color: pale brown (RAL8025), with postmedial line lighter in color, darker terra brown patch at apex. *Hindwing dorsum*: homogenous terra brown coloration, slightly lighter than forewing. Medially, a curved transverse line lighter brown. *Hindwing ventrum*: Dark terra brown with a pale brown transverse line. *Genitalia* (Figure 2).

Uncus strongly sclerotized, with wide base and single bent hook-like apex. Base of uncus with a pair of large socii made of a two folded sclerotized sheet bordered with numerous strong teeth. Valves elongated, not reaching the uncus, rather narrow, with apex rounded and a strong sclerotized spine at the dorsal base of the valve. Aedeagus short and cylindrical, with no teeth or cornuti. Caecum penis present. Vesica with a ventral diverticulum. No cornuti on vesica.

**Figure 4.**
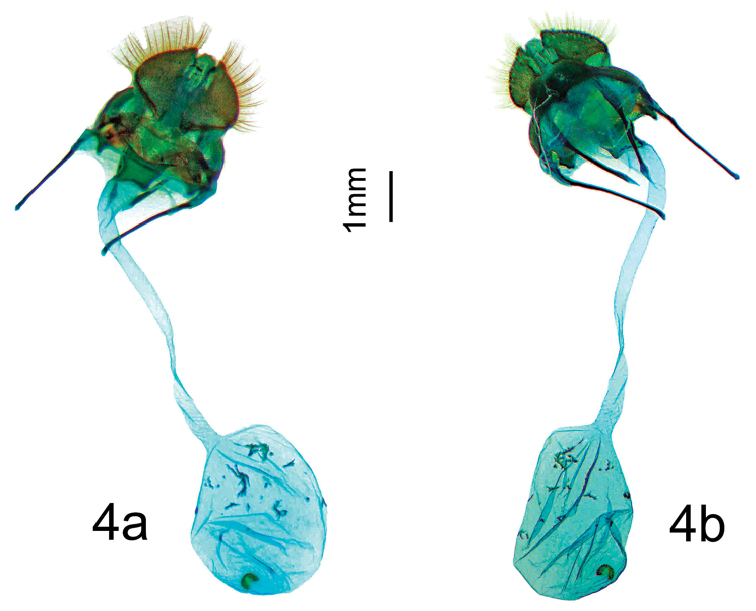
*Apatelodesnavarroi* sp. n. female paratype genitalia (H1386): **a** Ventral view **b** Dorsal view.

***Female.****Antennae*: similar to male but rami shorter. *Head*: Similar to male but labial palpi thinner and shorter, not reaching front. *Thorax*, *Legs*: As in male. *Forewing dorsum*: Forewing length: 22 mm, wingspan: 42 mm. Similar to male but broader, apex slightly more falcate, and termen below apex more concave. *Forewing ventrum*, *hindwing dorsum*, *hindwing ventrum*: As in male. *Genitalia* (Figure 4).

Papillae anales hemispherical, slightly bulbous and covered with setae. Apophyses thin and cylindrical, slightly spatulate at tip, anteriores about the same length as posteriores. Ductus bursae as a very long (about 5 mm, twice the length of corpus bursae), narrow, sinuous ribbon, of equal diameter for entire length. Ductus bursae sclerotized at entry near ostium bursae. Bursa copulatrix smooth and ovoid with a horse-shoe shaped, semi circular, single signum at extremity of bursa, signum equipped with minute teeth, inward pointing.

#### Distribution.

*A.navarroi* sp. n. is presently only known from Argentina, Jujuy, at medium altitude.

### Apatelodes chalupae sp. n.

Taxon classificationAnimaliaLepidopteraBombycoidea

http://zoobank.org/CF2AE03B-75CD-4791-9BF4-8C6DA342EF4D

Figs 5 , 6

#### Type.

**Figure 5.**
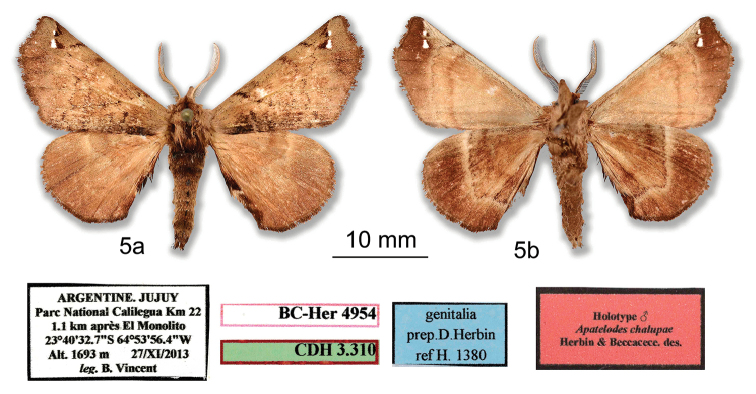
*Apatelodeschalupae* sp. n. male holotype: **a** Dorsal view **b** Ventral view.

**Figure 6.**
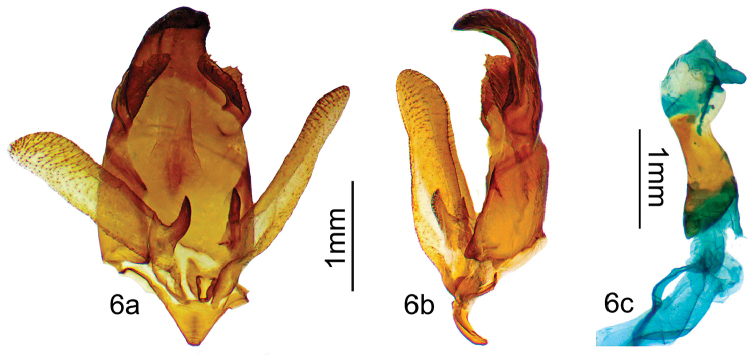
*Apatelodeschalupae* sp. n. male holotype genitalia: **a** Ventral view **b** Lateral view **c** Phallus lateral view.

***Holotype male.* ARGENTINA: Jujuy**: Parc National Calilegua Km 22, 1.1 km après El Monolito, 23°40'32.7"S; 64°53'56.4"W, Alt. 1693m, 27/XI/2013, *leg.* B. Vincent / D. Herbin genitalia prep. H1380/ HOLOTYPE ♂ *Apatelodeschalupae* Herbin & Beccacece des. / CDH 3.310 / BC-Her4954. (Figs 5, 6). *In*MNHN. No paratypes.

#### Etymology.

*Apatelodeschalupae* is named after Dr. Adriana Chalup (IFML), Geometridae and Noctuidae lepidopterist specialist.

#### Diagnosis.

*Apatelodeschalupae* sp. n. is similar to *A.navarroi* sp. n., but is much lighter in color (see comparison in Figs 10–11), bears two small hyaline spots near apex bordered proximally by black scales. The male genitalia also show structures similar to those of *A.navarroi* sp. n., but reduced in size, and with less developed socii, including less developed teeth bordering the socii. A distance in COI barcodes of 4.3% is found between these two species, as shown in the tree in Figure 16.

#### Description.

***Male.****Antennae*: Bipectinate to the tip. Scape, pedicel and antennomeres beige, rami beige brown. *Head*: beige brown, labial palpi thick, beige brown, slightly ascending and reaching beyond the front. Eyes naked, dark brown. *Thorax*: ochre brown with longitudinal black central line. *Legs*: beige brown, tibia appearing thick due to long beige brown scales. *Forewing dorsum*: Forewing length: 16 mm, wingspan: 32 mm. Triangular, apex acute, outer margin slightly concave below apex. Ground color light ochre brown (RAL8001), maculation similar to previous species with two small hyaline spots near apex, bordered proximally by tiny black marking. A black comma-shaped mark exists between costa and the smaller hyaline spot. A dark brown diffuse marking present basally in antemedian area. Antemedial and postmedial lines faint. *Forewing ventrum*: Ground color beige (RAL1001). Outer margin copper brown (RAL8004). Postmedial line light brown. *Hindwing dorsum*: coloration uniform light ochre brown, a faint lighter beige longitudinal line present. *Hindwing ventrum*: coloration rather uniform copper brown with contrasting beige distal longitudinal line inwardly bent at CuA2 and diffuse brown proximal longitudinal line. *Abdomen*: a black collar at interface of thorax/abdomen. First two abdominal segments dorsally reddish brown, remaining segments ochre brown. Black spot present dorsally on each abdominal segment. *Genitalia* (Figure 6).

Uncus heavily sclerotized, wide, bent mesally, with a simple hooked apex. Base of uncus with a pair of medium size socii made of a two folded sclerotized sheet bordered with few small teeth. Valves elongated, rather narrow, not reaching the uncus, with apex rounded and a strongly sclerotized spine at the dorsal base of the valve. Aedeagus short and cylindrical, with no teeth or cornuti. Caecum penis present. Vesica with a ventral finger like diverticulum and a smaller lateral diverticulum. No cornuti on vesica.

***Female.*** Unknown.

#### Distribution.

*A.chalupae* sp. n. is only known from the type locality in Argentina.

#### Remarks.

Initially, we thought that the type specimen of *A.chalupae* sp. n. was a lighter colored representative of *A.navarroi* sp. n., however the COI barcodes showed a significant distance between the two species (4.3%), further confirmed by the differences found in the male genitalia.

**Figure 7.**
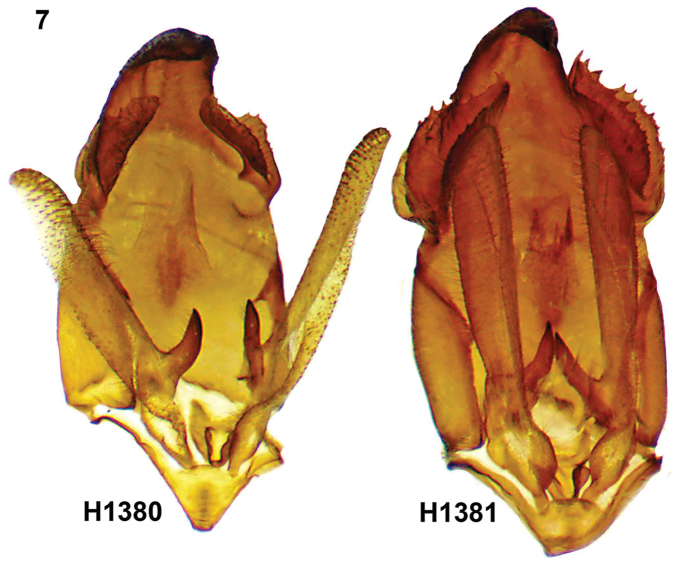
Comparative views of male genitalia: Left: *Apatelodeschalupae* (H1380) Right: *Apatelodesnavarroi* (H1381).

Figure 7 presents a side-by-side comparison between the genitalia of both species, from a photo taken under a microscope with both male genitalia taken in the same picture, therefore at the same scale. Smaller genitalia size for *A.chalupae* sp. n., the most striking difference is in the relative size of the socii, and the much stronger teeth present on the edge in *A.navarroi* sp. n.

In Figure 16, we present the tree built with MEGA6, with the new taxa *A.navarroi* sp. n., *A.chalupae* sp. n., and the most similar previously described species: *A.schreiteri*.

### Apatelodes ulfi sp. n.

Taxon classificationAnimaliaLepidopteraBombycoidea

http://zoobank.org/628182A5-B35C-470B-A16D-00B2156E5E2A

Figs 8 , 9

#### Types.

**Figure 8.**
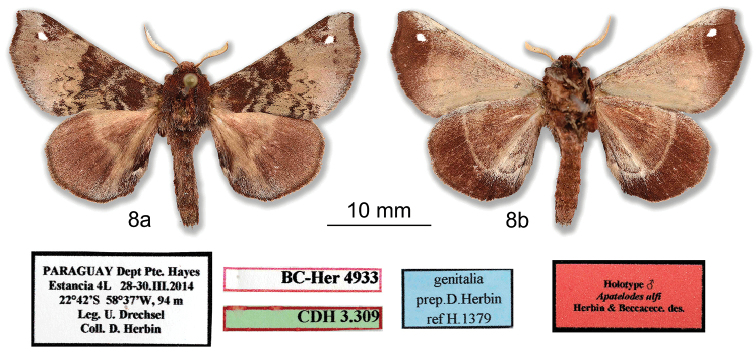
*Apatelodesulfi* sp. n. male holotype: **a** Dorsal view **b** Ventral view.

**Figure 9.**
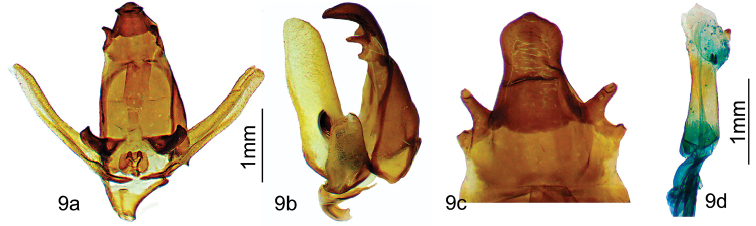
*Apatelodesulfi* sp. n. male holotype genitalia: **a** Ventral view **b** Lateral view **c** Uncus and socii details (ventral view) **d** Phallus lateral view.

***Holotype male.* PARAGUAY: Dept Presidente Hayes**: Estancia 4L, 28-30.III.2014, 22°42'S; 58°37'W, 94m, *leg.* U. Drechsel, Coll. D. Herbin / genitalia prep.D. Herbin H1379 / HOLOTYPE ♂ *Apatelodesulfi* Herbin & Beccacece des. / CDH 3.309 / BC-Her4933. (Figs 8, 9). *In*MNHN.

***Paratypes*** (14 males total): **BOLIVIA**: 1 male. **Dept Tarija**: Camatindi à Capirenda km 16, 493 m, 07.XI.2007, 21°01'07"S; 63°15'51"W, *leg.* Barbut, Vincent & Levêque/ genitalia prep.D. Herbin H830/ CDH 3.321/ BC-Her1918. *In*CDH; **BRAZIL**: 1 male. **Mato Grosso do Sul**: Environs de Rio Brilhante, Fazenda Senhor João Brandão, III.1966, *leg.* Cl. Moinier, Collection Jacques Plante. *In*MHNG; 1 male. **Mato Grosso do Sul**: Environs de Rio Brilhante, Fazenda Senhor João Brandão, III.1966, *leg.* Cl. Moinier, Collection Jacques Plante/ genitalia prep.D. Herbin H1423. *In*MHNG; **PARAGUAY**: 1 male. **Dept Presidente Hayes**: Estancia Tendota, 28-30.III.2014, 25°00'S; 58°05'W, 80m, *leg.* U. Drechsel, coll. D. Herbin/ CDH 3.317. *In*CDH; 2 males. **Dept Concepción**: Garay Cue, 04-09.VI.2013, 22°42'S; 57°22'W, 212m, *leg.* U. Drechsel, Coll. D. Herbin/ CDH 3.313 and CDH 2.803. *In*CDH; 1 male. **Dept Concepción**: Garay Cue, 25-29.IV.2013, 22°42'S; 57°22'W, 212m, *leg.* U. Drechsel, coll. D. Herbin/ CDH 3.318. *In*CDH; 1 male. **Dept Concepción**: Garay Cue, 27-30.IX.2014, 22°42'S; 57°22'W, 212m, *leg.* U. Drechsel, coll. D. Herbin/ CDH 3.319. *In*CDH; 3 males. **Dept Canindeyú**: 15-17.III.2016, 24°08'S; 55°31'W, 195m, *leg.* U. Drechsel, coll. D. Herbin/ CDH 3.312, CHD 3.314 and CDH 3.315. *In*CDH; 2 males. **Dept Boquerón**: Aurora Chaquena, 02.V.2015, 22°44'S; 60°00'W, 212m, *leg.* U. Drechsel, coll. D. Herbin/ CDH 3.316 and CDH 3.320. *In*CDH; **ARGENTINA**: 1 male. **Salta**: RN50 a Isla de Cañas Km31, 04.XII.2013, 23°04'06"S; 64°33'29.8"W, 547m, *leg.* B. Vincent/ CDH 3.323/ BC-Her4936. *In*CDH;

#### Etymology.

*Apatelodesulfi* sp. n. is named after Ulf Drechsel in Paraguay, who collected the majority of the known specimens.

#### Diagnosis.

*Apatelodesulfi* sp. n. is a rather small species, with the basal half of the forewing *dorsum* dark reddish brown very contrasting with the light colored (grey beige) postmedial and marginal area. A single hyaline preapical spot. The *ventrum* with inverted contrasting area compared to *dorsum*: darker on the marginal area, and lighter in median and basal area. The particular feature of the male genitalia lies in the socii, showing two ventral projections, one very short and truncated, another slightly longer.

#### Description.

***Male.****Antennae*: Bipectinate to the tip. Scape, pedicel, antennomeres and rami beige. *Head*: brown red (RAL3011) with some beige-tipped scales, labial palpi thick, brown, projected forward, eyes dark brown. *Thorax*: prothoracic collar brown red, thorax vinaceous red (RAL3005). *Legs*: Tibia thick with long hair like scales, brown and whitish for prothoracic legs, brown red for mesothoracic and metathoracic legs. *Abdomen*: brown red. *Forewing dorsum*: Forewing length (n = 13): 13–17 mm, wingspan: 28–35 mm (holotype: length 16 mm and wingspan 34 mm). Ground coloration grey beige (RAL1019), with basal half oxide red (RAL3009) contrasting with two grey beige undulating antemedial lines. Single elliptic hyaline spot, bordered proximally with an oxide red small triangle, distally by a small oxide red spot present near apex. Termen bordered with narrow oxide red. Postmedial line crenulated, oxide red. *Forewing ventrum*: Ground color beige (RAL1001) with some oxide red scales near costa. Marginal area oxide red with beige triangle above hyaline spot. *Hindwing dorsum*: Ground color red brown (RAL8012) with beige median line. Termen bordered by nut brown (RAL8011). *Hindwing ventrum*: Marginal area oxide red with beige postmedial line, antemedial and medial areas of lighter color due to beige scales interlaced with oxide red scales. *Genitalia* (Figure 9):

Uncus downcurved, strongly sclerotized, wide, with a single small spine at apex. At base of uncus, socii exhibit two finger-like extensions, one short and truncated and one longer (see Figure 9c). Valves elongated, rather narrow, with apex rounded. Process at base of valve strongly sclerotized with two small apical teeth. Aedeagus short and cylindrical, caecum penis present, with no teeth or cornuti. Vesica with a small ventral finger like diverticulum, then a very small diverticulum laterally, slightly sclerotized (appearing orange in Figure 9d).

***Female.*** Unknown.

#### Distribution.

Specimens of *A.ulfi* sp. n. have been collected in various localities in northern Argentina, southern Bolivia, central western Brazil and north to south Paraguay. Possible extension of the range to Peru remains to be investigated.

#### Remarks.

DNA barcoding of specimens from various localities reveal that these populations are all perfectly aligned (i.e. 0% distance between specimens of northern Argentina, southern Bolivia and Paraguay. Specimens from Brazil in MHNG not barcoded) despite some variation in wingspan.

A similar specimen in CDH, from northern Bolivia (Nor Yungas, Coroico area), shows a larger size and some differences in habitus, and has therefore not been included in paratype series. Similar specimens are likely to be found in southern or central Peru, this will then enable to verify identity or not with *A.ulfi* sp. n.

### Additional taxonomic notes

***Note 1***: A comparison of species similar to *A.schreiteri*.

**Figures 10–12.**
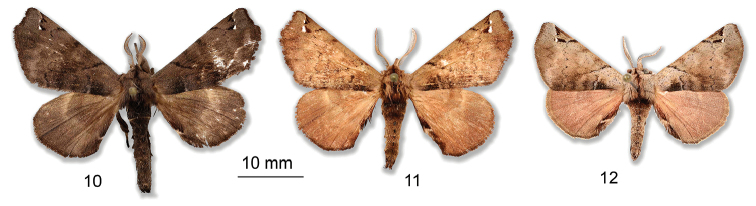
Comparison of similar *Apatelodes* species from the southern Neotropical region: **10***A.navarroi* sp. n., male **11***A.chalupae* sp. n., male **12***A.schreiteri*, male.

As illustrated in Figures 10–12, where a specimen of *A.schreiteri* is figured together with *A.navarroi* sp. n. and *A.chalupae* sp. n., differences in size of the specimens is obvious. The figured *A.schreiteri* specimen (Figure 12) was also collected in Argentina, Jujuy, Parc Calilegua, 1028 m (specimen CDH3.334, barcode BC-Her4940, barcoded and belonging to the *A.schreiteri* clade shown in Figure 16). Apart from the smaller size of this species compared to *A.navarroi* sp. n. and *A.chalupae* sp. n., the shape of the forewing in *A.schreiteri* is such that the termen is rather rounded, without the truncated apex found in the two newly described species. A significant difference also lies in the black marking proximal to the larger hyaline spot: in *A.schreiteri* this marking is more elongated than in the other two species.

**Figures 13–14.**
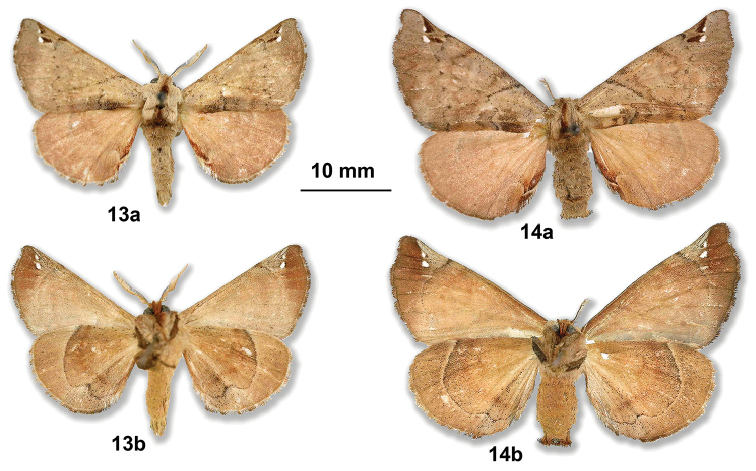
**13***A.schreiteri* male syntype (type n° 26354 USNM) **a** dorsal view **b** ventral view **14***A.florisa* female holotype (type n° 34696 USNM) **a** dorsal view **b** ventral view (photos D. Herbin, courtesy USNM).

Re-examination of the types in the USNM reveals exactly the same configuration of moth size, termen, and black markings in the syntype male of *A.schreiteri*, and in the female holotype of *A.florisa* Schaus, 1939. Both type specimens originate from Argentina, Tucumán. Figures 13–14 present the holotypes of both *A.schreiteri* and *A.florisa*: the female is slightly larger than the male, as observed in all other Apatelodidae species. We can observe the exact identical configuration of the forewing dark markings and hyaline spot in the male and female, this being generally a key in all species of *Apatelodes* to correctly allocate a female to the corresponding male.

We therefore consider that *A.florisa* Schaus is a subjective junior synonym of *A.schreiteri* Schaus and propose: *Apatelodesflorisa* Schaus, 1929 = *Apatelodesschreiteri* Schaus, 1924, syn. n.

Note: the genitalia of these holotypes have not been examined, but being the case of a male compared to a female this would not have actually helped in demonstration.

***Note 2***: The examination of various primary types enables the recognition of an anomaly in the present classification of Bombycoidea: *Cartharabrunnea* Dognin, 1916 (holotype male examined in USNM) was previously placed in the genus *Quentalia* Schaus, 1929 (Becker 1996), and more recently with the same combination in the global Bombycoidea checklist (Kitching et al. 2018). The genus *Quentalia* being now included in Bombycidae (Zwick 2011, Kitching et al. 2018) and separated from Apatelodidae, this species should instead be classified in the apatelodid genus *Apatelodes*.

**Figure 15.**
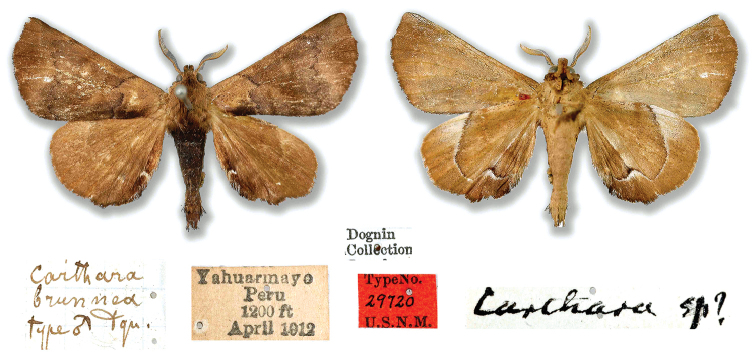
Holotype of *Cartharabrunnea* Dognin, 1916 (photos D. Herbin, courtesy USNM).

Figure 15 illustrates the type specimen of *Cartharabrunnea* (photo courtesy USNM), supporting the obvious proposed reclassification. The habitus, venation, antennae, and patterning, all suggest that this species belongs in Apatelodidae.

Draudt (1928: 681) established the new genus *Quentalia* and indicated: “This genus will contain the many species described under *Carthara* Wkr which was first used in Cat. Lep. Het. B.M. 33, p.914....”, and this is likely the root of the transfer of *brunnea* to *Quentalia*, as Dognin originally placed this species in *Carthara*.

The taxon *brunnea* is not addressed in the text nor in the color plates by Draudt (1928), but can be found in the same work under: Alphabetical List of the American Bombycidae on page 710 under *Q.brunnea*. It is likely that since Draudt (1928), no one has re-examined the holotype, and the mistaken classification has been propagated. Here, the following taxonomical rearrangement is proposed: *Cartharabrunnea* Dognin, 1916 = *Quentaliabrunnea* (Dognin, 1916) = *Apatelodesbrunnea* (Dognin, 1916), comb. n.

We notice a similarity of *A.brunnea* to the species newly described above, but no hyaline spot exists on the forewings in *A.brunnea*.

**Figure 16.**
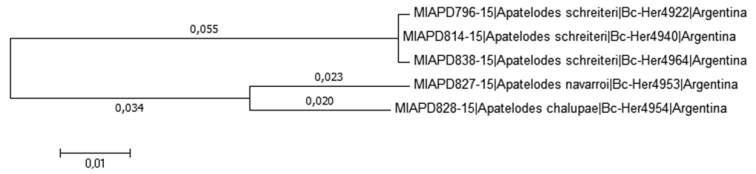
Phylogenetically inferred evolutionary relationships between related *Apatelodes*, using COI. The tree was constructed with the neighbor-joining method.

## Supplementary Material

XML Treatment for Apatelodes navarroi

XML Treatment for Apatelodes chalupae

XML Treatment for Apatelodes ulfi
